# Traits and skills of effective leaders in antimicrobial stewardship

**DOI:** 10.1017/ash.2023.499

**Published:** 2023-12-04

**Authors:** Tanaya Bhowmick, Ahmed Abdul Azim, Navaneeth Narayanan, Keith Kaye

**Affiliations:** 1 Department of Medicine, Division of Allergy, Immunology and Infectious Diseases, Rutgers Robert Wood Johnson Medical School, New Brunswick, NJ, USA; 2 Department of Pharmacy Practice and Administration & Center of Excellence in Pharmaceutical Translational Research and Education, Rutgers University Ernest Mario School of Pharmacy, Piscataway, NJ, USA

## Background

Although there is some guidance on implementation of an antimicrobial stewardship program^1^ and the knowledge and skills necessary for effective stewardship leadership,^2^ there is no published literature on effective leadership traits or strategies for a stewardship leader to be successful.

In this commentary, we discuss the differences between leadership and management, and skills and traits important for both roles to be effective. While leadership is the focus of this manuscript, leaders in stewardship often directly oversee and manage at least some components of the stewardship program, and therefore, effective leaders in stewardship should be familiar with skills and traits necessary for effective management. This document is meant to serve as a guide for both physician and pharmacy leaders to develop and strengthen themselves in these traits.

## Leadership vs management

One important concept regarding discussions about leadership is to differentiate leadership from management.^3^ Leadership is imagining the possibilities. A leader has a vision and determines what should be done and why the vision is important to accomplish. In addition, leaders set the culture and tone of the work environment and motivate the team to execute the work to make the vision a reality.

Management is execution. Managers determine how and when tasks are performed to achieve program goals. The manager oversees day-to-day operations and controls program structure and staffing.

Traits that have been attributed to effective leaders as well as to successful managers are necessary to effectively lead a successful program.

## Key traits to be an effective manager

Effective managers should be organized and develop a game plan to execute a vision. For example, a pharmacist manager for a stewardship program is tasked to develop an antibiotic restriction policy. The manager would select which antibiotics to restrict and determine who would approve the antibiotics along with the approval schedule. Furthermore, the manager would set up a plan in anticipation of roadblocks or difficulties, such as non-compliant physicians.

Managers oversee process. They establish the rules for the team members to follow. For example, managers might develop a standard operating procedure (SOP) with input from the team for routine activities, such as prospective audit and feedback. An SOP for this might include which drugs or disease states to target, how to obtain daily lists of cases to target, and documenting efforts and outcomes.

Effective managers are expected to direct day-to-day efforts. They must determine the resources necessary for effort execution and ensure those resources are available. When unexpected changes occur, a manager should strategize and plan accordingly to make sure that routine activities can continue. For example, if the stewardship pharmacist who approves restricted antimicrobials is out sick, an effective physician manager might devise an alternative plan, such as temporarily shifting approval responsibilities to another team member.

Managers should be people-focused and take care of their team. Not only should they be available to listen to their team’s concerns but they should also involve them in decision-making. A manager may decide to change certain processes based on constructive feedback from team members. For example, a stewardship pharmacist is getting berated by physician Z when not approving antibiotic X. The manager would need to be available to provide a “safe space” where the team member can broach the issue, receptive to hearing and learning about the issue, and empathetic to the position that the pharmacist is in. In addition, a good manager should take action to address the situation (e.g. by talking to physician Z and escalating to a supervisor if necessary).

## Key traits to be an effective leader

Effective leaders are visionaries.^4^ They know the current state of a program and have envisioned a brighter and better future. A stewardship example would be, after determining that the current program is under-resourced particularly considering future goals, proposing to add two stewardship pharmacists to the team over the next 5 years by developing a business plan and return on investment to secure the funding and then getting buy-in from hospital leadership (Table 1).

**Table 1.** Examples of management and leadership traits

Traits	Examples
*Management traits*	
Ability to execute	Implementation of antibiotic restriction policy—select antibiotics, determine approval structure, determine schedule, and anticipate difficulties with plan of action
Process management	Develop standard operating procedure prospective for audit and feedback
Ability to direct day-to-day efforts	Review necessary resources and anticipate changes that need to be made in routine and process
People focused	Consider needs of team members
*Leadership traits*	
Visionary	Envision possibilities to expand current state
Emotional intelligence	Awareness of own strengths/weaknesses and continued efforts for self-improvement
Honesty and integrity	Share positives and negatives directly and transparently with team members
Inspiration	Provide feedback and instill a sense of pride in team members
Communication skills	Keep team informed about current state, future plans, and obstacles
Ability to challenge	Change the status quo if it will lead to betterment (e.g., having ID physicians and pharmacists take stewardship call instead of fellows)

Good leaders should have emotional intelligence. They are self-aware of their own abilities and shortcomings, can self-regulate their own emotions, and are empathetic. The Dominance, Influence, Steadiness and Conscientiousness (DISC) profile tool^5^ can be used to learn about one’s own personality, as well as the personalities of others they work with. This type of knowledge can inform strategies to improve productivity of the team. Moreover, a self-directed process, such as the Pick, Apprise, Collect, and Elicit (PACE) model,^6^ can help a leader assess their own ability to lead. The earlier that a leader assesses their own skills and elicits feedback from team members, the more likely they will successfully address weaknesses and improve leadership skills.

Leaders should also value their team members’ efforts and in doing so, support and facilitate those efforts. For instance, if team members are spending a lot of time extracting and organizing data, a good leader would recognize that there is a lack of necessary infrastructure and might petition for a data analyst FTE to create and run reports so that stewardship team can focus more on patient care.

Good leaders inspire their team and help the team understand their roles in a bigger context. For example, an effective pharmacist stewardship leader might stress to team members that they contribute valuable effort that helps to prevent and improve the management of life-threatening infections due to resistant, hospital-acquired pathogens and in doing so, makes the stewardship team one of the most impactful committees in the hospital. Team members can take pride in their work, which contributes to job satisfaction and continued motivation.

Effective leaders should be honest and have integrity and transparency to garner the trust and support of their team. Leaders should always keep their team informed about the current state of affairs, planned future changes, and potential obstacles that might be encountered along the way.

Leaders challenge the status quo to solve problems and/or improve the current state of affairs, oftentimes by thinking outside the box. For example, a stewardship program may be utilizing ID fellows to manage antibiotic approvals because, in part, there has historically been resistance among faculty to changing this practice. Due to issues with fellow workload and concerns regarding the effectiveness of fellow-run approvals, a good physician leader might commit to changing the approval management process and having ID Pharmacists and attendings lead this effort, even though this might not be the most popular decision among peers.

## Tips for becoming an effective leader in stewardship

It is important for the leader to be familiar with the fundamentals of antimicrobial stewardship. This builds the leader’s credibility with the team. Moreover, the leader is in a better position to identify the best-suited individuals to fulfill different roles. An effective leader builds a team that has members with complementary knowledge, skill sets, experiences, and characteristics. Once built, good communication (e.g. regular check-ins) and positive reinforcement (e.g. giving feedback and recognition when appropriate) can maintain a productive and well-functioning team.

Strong relationships are a key component of being an effective leader. In addition to maintaining positive and productive relationships among team members, external relationships outside of the team are also important. The leader can build strategic relationships in their own institution with key decision makers, initially through introductory and exploratory meetings. In meetings, current efforts and accomplishments of the team can be showcased and possibilities for future services and production can be discussed (if necessary resources are provided). In addition, networking with colleagues at other institutions to share best practices or strategies to overcome challenges can result in the betterment of the program.

It’s important for leaders to remain clinically active and work under the rules set forth by their team. In doing so, leaders are working on common ground with their colleagues. This builds their credibility with other providers, which is particularly important when addressing outlier physicians (e.g. infectious diseases physician who prescribes off-label guidance of the newer niche agents when other effective therapies are available).

Lastly, a good leader fosters the development and growth of the team, giving credit to the team for accomplishments and accepting responsibility and blame for failure/problems. They should take an interest in the careers of team members and mentor them to help them grow individually and achieve success professionally.

In summary, to be an effective and successful leader in antimicrobial stewardship often requires both leadership and managerial skills. Because of the multi-disciplinary and diverse nature of stewardship activities, there are many opportunities for stewardship leaders to positively impact the careers of team members, the clinical course of patients, and their own personal growth.
